# Differentiation of Multiple Fluorescent Powders, Powder Transfer, and Effect on Mating in *Aedes aegypti* (Diptera: Culicidae)

**DOI:** 10.3390/insects11110727

**Published:** 2020-10-24

**Authors:** Diana Rojas-Araya, Barry W. Alto, Derek A. T. Cummings, Nathan D. Burkett-Cadena

**Affiliations:** 1 Florida Medical Entomology Laboratory, Department of Entomology and Nematology, IFAS, University of Florida, Vero Beach, FL 32962, USA; bwalto@ufl.edu (B.W.A.); nburkettcadena@ufl.edu (N.D.B.-C.); 2 Department of Biology and Emerging Pathogens Institute, University of Florida, Gainesville, FL 32610, USA; datc@ufl.edu

**Keywords:** marking techniques, mosquito, mating process and behavior, conspecific interactions

## Abstract

**Simple Summary:**

Fluorescent powders are one of the most used materials for externally marking mosquitoes, such as *Aedes aegypti*, a vector of numerous human pathogens. They can be used to explore multiple biological questions related to population size, dispersal, and other interactions related to the spread of mosquito-borne diseases. To ensure marking practical aspects and that these powders do not interfere with mosquito natural behaviors, differentiation of multiple colors applied externally in the same mosquito, their impact on mating, and their transference between individuals after copulation and same-sex interactions were studied. Multiple color differentiation was possible, except for green–yellow combination. No important effect of powder marking was found on any of the mosquito mating phases: coupling (recognition and genital contact), copulation (genitalia engagement and semen transfer), and insemination (deposition of the sperm in the female reservoirs). After copulation with a powder-treated mate, >80% of females and all males had powder in genitalia, legs, and wingtips. The transfer of powder, between same-sex individuals, occurred only in males. In general, fluorescent powders had a little observable effect on *Ae. aegypti* mating, suggesting that these markers do not alter this important vector life-history trait and are a useful and viable tool for mosquito studies.

**Abstract:**

Five different fluorescent powders (orange, yellow, green, blue, and violet) were tested on *Aedes aegypti* adults to evaluate the differentiation of multiple fluorescent powder colors applied externally in the same female mosquito, their effect on coupling time, copulation time, insemination success, mate choice, and the extent of transference of powders between marked and unmarked individuals, either during copulation or same-sex interactions. Marking with multiple powders was evaluated after applying different powders in the same female at different times and combinations. The comparative effect of powders on mating was explored using different cross-combinations of marked/unmarked couples. Transference of powders between marked/unmarked individuals after copulation was checked in mated individuals, and between same-sex interactions by allowing them to interact under crowded and uncrowded conditions. Identification of the colors included in multiple markings in the same individual was possible when exploring almost all combinations (exception: green–yellow). No important effect of powder marking between cross-combinations was found on coupling time (overall 95% CI (Confidence Interval) 37.6–49.6 min), copulation time (overall 95% CI 17–20 s), insemination success, nor their mate choice. Transferred powder after copulation activity, concentrated in genitalia, legs, and the tip of wings, occurred in >80% of females and 100% of males. Powder transference in legs and genitalia, between same-sex individuals, occurred only in males (ranged between 23–35%) under both density conditions. The lack of important effects of these powders on the studied aspects of *Ae. aegypti* provides information about their usefulness and limitations, which should be recognized for future applications and to avoid bias.

## 1. Introduction

A wide variety of methods and techniques for marking mosquitoes have been developed, many of which have been used to explore multiple biological questions related to population size and dynamics, dispersal, feeding behavior, trophic-level interactions, and other ecological interactions [1,2]. Among available markers, fluorescent powders are the most commonly used material for externally marking mosquitoes [1]. Several chemical options of these fine particles are available, with some designed to be free of formaldehyde, heavy metals, azo compounds, perfluorooctanoic acid, aromatic amines, bisphenol A (BPA), and polyaromatic hydrocarbons [3]. Apart from being inexpensive, available in different colors, easily applied, and detected without destroying the specimen for 30 days on marked females, under controlled laboratory and semi-field environments, previous experiments using marked *Aedes aegypti* (Linnaeus) females, have also revealed their low impact on survival of different age cohorts, female blood-feeding success, tethered flight velocity, and their recapture in baited traps [4,5]. Other applied questions remain unanswered, like potential difficulty in distinguishing individual markers when used in a multiple marker approach in which individuals are marked with more than one fluorescent powder at different times and combinations, their impact on mosquito mating, and their transference during copulation or same-sex interactions. Addressing these questions will improve our ability to reliably use fluorescent powder marking of individuals to further elucidate mosquito biology, especially in field settings, and develop novel control strategies that exploit mosquito biology.

Multiple marking of the same individual mosquito during different moments of their life span or activity periods, expand the practical uses of the fluorescent powders as marking tools. This technique allows the time determination of successive recaptures, that inserted in different types of studies, can be used for exploring population size, dispersal, survival, age structure composition, feeding behavior, and overcome limitations when working in low-density habitats [6]. Marking females with a single powder color is the most common approach used in mosquitoes, but some studies using *Drosophila* flies [7] or the chrysomelid *Diabrotica virgifera* [8], have used multiple marking colors, including applying one, two and three powder colors in the same adult. These approaches have advantages and limitations, the latter, principally the difficulty of being able to differentiate between more than two colors simultaneously.

To our knowledge, the effects of fluorescent powder marking on *Ae. aegypti* mating has not been studied in detail. Experiments exploring the effects of other variables on mating characteristics of *Ae. aegypti* males, such as male mating competitiveness [9] or the effect of fungal pathogen *Metarhizium anisopliae* infection on mating activity [10], have used powder marking to distinguish between treatment groups, but the marking status was used in both treated and control adults.

*Aedes aegypti* mating takes place naturally mainly in aerial swarms near the host [11,12,13] or between compatible mating pairs in laboratory settings [14,15], when males and females recognize and interact between each other through visual, acoustic and chemical cues [12,16,17]. The mating process consists of three phases, from recognition to genital contact (coupling), the “venter-to-venter” genitalia engagement and semen transfer (copulation), and the deposition of the spermatozoa and male secretions in the female sperm reservoirs or spermathecae (insemination) [18]. Duration of each mating phases varies, depending on factors such as the sexual maturation (males require 36 to 48 h to rotate their genitalia before mating) and receptiveness of each couple member, age, size [19], their flight behavior, and harmonic convergence [17], the presence of other males, the time it takes for the male to be in the correct position, and female resisting or cooperating behavior [16]. Reported copulation time (the period that elapses between the beginning of the copulation and its end) varies from 4 to 59 s, after which, with approx. 40 s of delay, sperm temporarily held in the bursa copulatrix, will travel into one to three spermathecae, completing their filling in 300 s [16,20,21]. Females typically mate only once, and sperm will remain viable during their entire reproductive life, packed and nourished in their spermathecae [16,22]. In contrast, a single male can copulate with multiple females during his lifespan and is able to copulate and inseminate successively with three to five females, after which sperm becomes depleted, and they need to replenish the seminal vesicles before additional mating [20,23,24].

Another experimental aspect of markings that can generate biased results is powder transference. Markers can be transferred from intentionally marked individuals to unmarked individuals when mosquitos interact inside collecting devices or in field conditions. This has been considered unlikely when aspirating mosquitoes in groups, and not observed when collecting mosquitoes with CDC (Centers for Disease Control and Prevention) light traps, or when aspirating mosquitoes individually or using a battery-powered aspirator [25]. Other experiments have explored transference among marked and unmarked individuals, without specifying in their methodologies if they used same-sex individuals or different sexes. For example, *Psorophora* mosquitoes heavily marked with fluorescent powder can transfer it to 1–3% of unmarked individuals in a confined space [26], but no powder transfer occurred between marked *Culicoides* biting midges and unmarked controls nor to the environment in which the marked individuals were held for a 24 h period [27]. Powder transference between different sex individuals and during copulation has been observed when placing equal numbers of *Drosophila pseudoobscura* dusted nonvirgin males and females; some genital transfer if nonvirgin individuals were allowed to clean themselves before mixing them under moderately crowded conditions; and no transfer during mating of virgin adults [6]. Studies using *Anopheles arabiensis* found that males are capable of transferring the mark, only detectable when examined under a stereoscope, to both males and females and that this transference increased over time [28], but no powder transference among adult mosquito mating pairs was found using *Culex quinquefasciatus* marked males and unmarked females [29]. Transference between marked males to unmarked females was also reported when using fluorescent powders in *Ae. aegypti*, as a tool for distinguishing between treatment and control groups [10].

From a practical perspective, the evaluation of multiple marking and powder transference between individuals is crucial for validating their use and recognizing their limitations or potential bias. From a biological point of view, understanding their impact on the mosquito mating process and their mate choice is essential, since the conclusions of any study using this tool, should be aware of their potential impact on male and female mating interactions. In this study, five different Day-Glo^®^ ECO Series [3] fluorescent powders were tested on *Ae. aegypti* adults to evaluate the differentiation of multiple fluorescent powder colors applied topically in the same female mosquito, their effect on coupling time, copulation time, insemination success, and mate choice, along with their transference between marked and unmarked individuals, either during copulation or same-sex interactions. The selection of these powders is related to their performance in previous experiments [4,5].

## 2. Materials and Methods

Five different water-insoluble fluorescent powders, with a specific gravity of 1.2 and a mean particle size of 4.5 µm, of the brand Day-Glo^®^ ECO Series [3] were tested on *Ae. aegypti* adults to evaluate their differentiation in the same individual after being marked with more than one powder, their effect on the mosquito mating process and mate choice, and their transference between marked and unmarked individuals. The experimental codes of the fluorescent powders (color-powder identification) used were: DG1 (fire orange—ECO 14), DG2 (Saturn yellow—ECO 17), DG3 (signal green—ECO 18), DG4 (horizon blue—ECO 19), and DG5 (ultra violet—ECO 20).

Protocols for rearing and marking *Ae. aegypti* adults (Monroe County Key West Florida strain, F23 progeny) were performed as previously described [5]. Briefly, adults were exposed to a fluorescent powder-rich environment inside paperboard cages (H10 × top D10 × bottom D7 cm) with mesh tops, by expulsing from a syringe (3 mL with hypodermic needle 26G (0.45 mm)) 0.5 mL of each powder (0.17 ± 0.01 g of powder, N:50) [30,31]. Presence of powder in the body of the mosquito was confirmed using a stereo microscope and a UV (Ultraviolet) light source (LED (Light-emitting diode) 395 nm ultraviolet flashlight), and through a maximum 5 points rubric score reached after adding a “1” following the confirmation of the presence of the powder in each mosquito body part (head, thorax, abdomen, wings or legs) [32].

Individuals from two control groups were used. One control group had females that were not handled or exposed to any powder (control colony: CC), and the other sham control group had females exposed to the marking technique but without using any powder (control application method: CAM). Different combinations of unmarked female (UF), marked female (MF), unmarked male (UM), and marked male (MM) were used.

### 2.1. Differentiation of Multiple Marking in the Same Individual

To investigate the influence of the application time and the recognition of two different powders in the same female, groups of 10 females were exposed to one fluorescent powder at time 0 h, and then independently to a second powder at four twelve-hour intervals (12, 24, 36, 48). Three replicates of twenty color combinations, exploring different color orders and time of application for all 5 powders, were performed. After the application of the second powder, females were collected, frozen, and inspected, in order to establish if both powders can be recognized separately in the same female and to give them a score.

To study more than two powders in the same female, three replicates of the two combinations were explored. Females were exposed to one powder at time 0 h, were re-exposed to a second powder at *t* = 24 h, a third powder at *t* = 48 h, a fourth powder at *t* = 72 h, and a fifth powder at *t* = 96 h. In this approach, 35 females were used per combination, and one hour after each application, 5 randomly selected females were collected, frozen, and analyzed in order to establish if sets of powders can be separately recognized in the same female. The order of colors in the two combinations was established by using the color wheel: one combination was in a clockwise direction (yellow–orange–violet–blue and green), and the other in the counter-clockwise direction.

### 2.2. Effect of Fluorescent Powders on Ae. aegypti Mating Process and Mate Choice

Different cross-combinations of marked/unmarked males and females were used to analyze the effect of fluorescent powders on *Ae. aegypti* mating process (coupling time, copulation time, and insemination success), and mate choice. The same rearing protocol described previously [5] was followed, but because body size influences the adult mating capacity, larval conditions were rigorously standardized in order to obtain large size specimens, by transferring 100 first instar larvae to each tray (W30 × D25.5 × H4 cm) containing 1 L of water and feed in a daily regimen of 75, 38, 75, 113, 150 mg food (1:1 yeast: lactalbumin) on days: 1, 3, 4, 5, and 6, respectively [19].

The comparative effect of fluorescent powders on the mating process of *Ae. aegypti* along the cross-combinations was explored by exposing one virgin male with up to 3 consecutive virgin females [10,20]. Adult virginity was accomplished by separating pupae individually into 15 mL vials containing 3 mL of water and confirmation of adult sex before setting up the experiments [19]. Twenty replicates, using 5–18 days old virgin mosquitoes, of five cross-combinations were explored: MM-MF, MM-UF, UM-MF, UM-UF_CAM, UM-UF_CC. Individuals were exposed to the application method, and to the powder or not (depending on if they are marked or unmarked) 24 h prior to the mating process experiment. Combinations that included one MF or MM with an unmarked conspecific, were marked using DG1 (orange); and those combinations in which both members of the couple were marked, DG1 and DG2 (yellow) were used. Each male was placed individually inside a 0.5 L rectangular prism transparent plastic bottle (W6 × D6 × H15 cm). The bottle was closed with their own modified lid, that had a hole covered with a hollow latex cylinder, and a piece of cotton wool that prevented mosquitoes from escaping. The five copulation combination bottles were placed inside a copulation arena, made with a white corrugated polypropylene sheet, that allowed the simultaneous time control and video recording of all combinations within each replicate. After the placement of the male inside the bottle, up to three virgin female mosquitoes were proposed in rapid succession, in which each female was removed with a mouth aspirator immediately after copulation, then the next female was added, and the bottle was gently shaken to stimulate flight [10,20]. The time taken by a male to initiate copulation with each female (coupling time—min) and the copulation time (s) was timed with a XREXS 4 channels digital timer clock and video—recorded with a mobile phone (Apple iPhone 6S) (Apple Inc., Cupertino, CA, USA), for 100 min maximum. For replacing the female, copulation was considered as the engagement of the genitalia for at least 10 s [16,20], but all couples were allowed to copulate without being disturbed and only after copulation was finished, the female was replaced. Each copulated female was placed individually for 48 h [20] in a separate paperboard cage with mesh tops (H10 × top D10 × bottom D7 cm) supplied with 10% sucrose moisturized cotton wool renewed daily. Then, they were anesthetized with ethyl acetate (for 10 s) and immediately inspected and scored. For the evaluation of the male insemination success, the female insemination status was checked by pulling out their ovipositor with the internal appendages and removing the spermathecae from the eighth abdominal segment in water under a stereo microscope [33]. The movement of the sperm was immediately checked in each of their three spermathecae, by placing them in between a glass slide and a coverslip (a thin piece of cotton was used on one of the edges, to prevent the spermatheca from bursting), and using 4× and 10× objective lens of a compound microscope (Olympus BH2 Series) (Olympus Corporation of the Americas, Center Valley, PA, USA). The number and position (central, and/or one or two of the lateral) of spermatheca with sperm was registered [34], and a filled spermatheca was recorded even if a low quantity of sperm was visualized.

To investigate if the presence or absence of fluorescent powders in females affects the male sexual mate choice, 5 unmarked virgin males were placed simultaneously with 10 marked and 10 unmarked virgin females inside a translucent polyester mesh cage (W32.5 × D32.5 × H32.5 cm) for 24 h. After this period, males were removed, and females were left inside the cage for 24 h more [20]. Then, they were anesthetized, inspected, scored, and checked for their insemination status, as previously described. Six replicates of this experiment were performed, 3 with DG1 and 3 with DG2. To evaluate powder transference related to copulation, the powder score was analyzed only for females that were inseminated, since they are the only ones for which a copulation interaction can be evidenced.

Mosquitoes used in both experiments were stored into 1.5 mL Eppendorf tubes and frozen for wing length measurements. The wings were removed under a stereo microscope and then mounted on microscope slides using double-sided tape. Wing digital images and measures, from the auxiliary incision to the apical margin excluding the fringe [35], were taken using a Meiji Techno optical microscope (Meiji Techno America, San Jose, CA, USA) (4×) and an AmScope MU 300 series camera and software [36].

### 2.3. Transference of Fluorescent Powders between Individuals during Copulation and Same-Sex Interactions

The transference of powders between individuals during copulation was studied using the marked/unmarked males and females from the different cross-combinations of the mating process and mate choice experiments. Their powder score, complemented with the description of the part of the mosquito body where it was observed, was recorded, and analyzed.

The transference of fluorescent powders between same-sex individuals was tested by allowing them to interact under two density conditions: crowded and not crowded. Three replicates of four different combinations of 3–4 day old marked (marked 24 h before the interaction) and unmarked females and males (MF-UF_CAM, MF-MF, MM-UM_CAM, MM-MM), were placed inside paperboard cages with mesh tops (H10 × top D10 × bottom D7 cm) in equal ratios, with a total number of 20 (not crowded conditions: NC) and 100 individuals (crowded conditions: C), respectively. Twenty-four hours after exposure, all specimens were frozen, individually examined, and scored. The same selection of powders was used as already described in the mating process experiment.

### 2.4. Statistical Analysis

Statistical analyses were performed in IBM SPSS Statistics [37]. A two-sided Fisher’s exact test was used to examine if the proportion of inseminated or non-inseminated females were the same among cross-combinations (mating process experiment) or among marked and unmarked females (mate choice experiment), and to test if the proportion of females or males where powder transference occurred was the same among combinations on both crowded or not crowded conditions (same-sex individuals powder transference experiment) or among cross-combinations 1-MM-MF and 2-MM-UF (mating process experiment). The same test was also used to compare the proportions of inseminated marked or unmarked females with 1, 2, or 3 filled spermathecae in the mate choice experiment. Follow-up multiple proportions comparisons were conducted, when significant effects were found, adjusting the *p*-values using the Bonferroni method [38]. For all analyses, an alpha level of 0.05 was used.

For the mating process experiment, a time-to-event analysis (based on the Cox proportional hazards model) was used for analyzing the coupling time or length of time until the occurrence of copulation events after each female was introduced to the male. One hundred minutes was used as the endpoint time of observation, the different cross-combinations as the factor of comparison, and age, wing size, and female sequential order of presentation to the male as covariates. Information on mosquito couples that had not copulated by the end of the observation period was censored. One-way analysis of variance (ANOVA) was conducted to compare, both mosquito females and males, wing size (mm) among cross-combinations. Using only the information of males that copulated with three females (N:25), a profile analysis, with one within-subjects factor (i.e., copulation time of a male with 3 sequential females) and one between-subjects factor (i.e., cross-combination groups) was conducted to examine if the copulation time (s) of each male, across all cross-combinations, varied after the successive copula with three sequentially presented females. A linear least-squares regression was used to predict the effect of the different cross-combinations on the number of filled spermathecae in inseminated females. The same type of regression was also used to assess if the score obtained in the females that had transferred powder after copulation was affected by the cross-combinations 1 (MM-MF) and 2 (MM-UF), age and size of the females and males, and the female order in which they were presented to the same male (first, second or third) [38].

## 3. Results

### 3.1. Differentiation of Multiple Marking in the Same Individual

The distinction of more than two colors in the same female, required the use of magnification (stereo microscope). Determination of color when a single color is used can be done accurately and easily with the naked eye or using UV light.

To analyze the effect of application time and the recognition of two different powders in the same marked female, 600 total females were analyzed. The combinations of yellow–orange, green–orange, and blue–orange were recognizable and all tested females had both powders in all their five body parts (score of five), regardless of the order of application and time elapsed between applications. The easiest combination to distinguish was when using orange and yellow together. Combinations of blue–yellow and blue–green showed similar results as the latter, with the difference that when the powder blue was applied second, its visualization was more difficult and only aided by the clustering of the powder in a body region. Regarding all the previous combinations, the pigment that was placed first usually remained in greater coverage and therefore was more visible than the second pigment. Irrespective of the application time or order, the combination of green–yellow was not distinguishable. The use of violet was also hard to identify and differentiate from the mosquito scales, which seem violet when observed under the UV light. Blue and violet powders look duller when compared to the other brighter colored powders.

To study more than two powders in the same female, 150 females total, grouped in two combinations, were analyzed. Recognition of each powder, after multiple powders application, was similar to what was observed when two of them were applied. Combinations of orange–yellow and green–blue (at 24 h) were recognizable, but the third powder (violet) at 48 h was not visible. The application of a fourth powder was feasible, but easier in the case of orange than in the case of blue. The application of the fifth powder (green or yellow) presented the same difficulty previously expressed, that is that the combination yellow–green, regardless of the order of application, is not visually distinguishable. All the females marked on multiple occasions with powders that were distinguishable, presented a score of five, irrespective of the application time or order.

### 3.2. Effect of Fluorescent Powders on Ae. aegypti Mating Process and Mate Choice

#### 3.2.1. Mating Process

One hundred males were used for the analysis of the effect of fluorescent powders on the *Ae. aegypti* mating process (coupling time, copulation time, and insemination success). Thirty-seven males did not copulate with any female by the end of the exposure time, 19 with one, 19 with two, and 25 with three females. Since the consecutive female (second or third) was presented to the same male after he copulated with the previous one, not all 300 available females were used, and only 207 were exposed to a male. Of this number, 132 females did copulate with the male, and 75 did not copulate by the end of the observation time. The frequency of copulation events was similar among mosquitoes of different ages (from 5 to 18 days old). Mean wing size of males (2.06 ± 0.06 mm) (F_4,93_ = 1.518, *p* = 0.263) and females (2.74 ± 0.15 mm) (F_4,123_ = 1.831, *p* = 0.127) were not significant different between cross-combinations.

**Figure 1:**
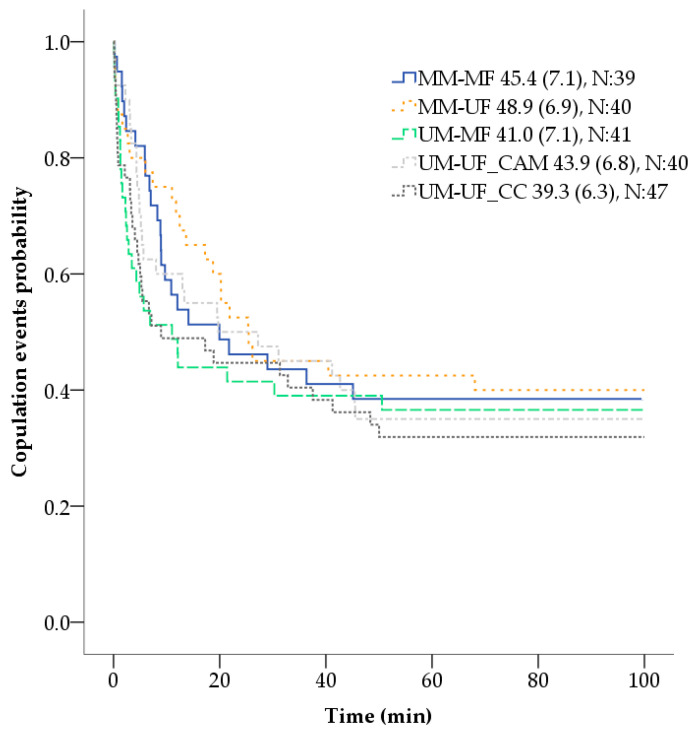
Coupling time (min) or length of time until the occurrence of copulation events after each female was introduced to the male of different cross-combinations. Numbers following the cross-combinations indicate mean (±SE) coupling time (min), and the total number of females sampled (N) per combination. Unmarked female (UF) marked female (MF), unmarked male (UM), marked male (MM), control colony (CC), and control application method-sham (CAM).

Coupling time (min) or length of time until the occurrence of copulation events after each female was introduced to the male was no different among cross-combinations (X^2^ = 2.36, *p* = 0.666, N = 207) (Figure 1). Overall coupling time CI (confidence interval 95%) was 37.6–49.6 min, and specific cross-combinations point estimates and CI were: MM-MF 45.4 (31.6–59.3), MM-UF 48.9 (35.4–62.3), UM-MF 41.0 (27.1–55.0), UM-UF_CAM 43.9 (30.7–57.2), and UM-UF_CC 39.3 (27.0–51.7) min. Individuals that did not copulate by the end of the observation time (100 min) were censored.

**Figure 2:**
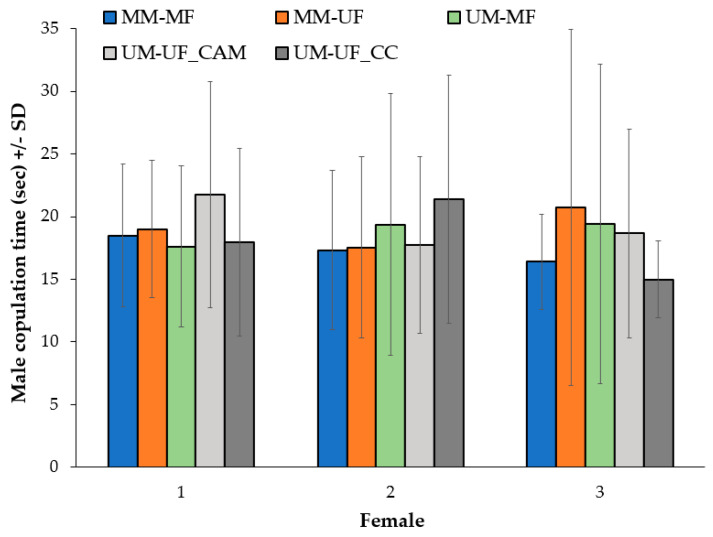
Male copulation time (s) (±SD) (male with three sequentially presented females) of all cross-combinations. Unmarked female (UF), marked female (MF), unmarked male (UM), marked male (MM), control colony (CC), and control application method-sham (CAM).

The presence or absence of powder in the different cross-combinations and the order of female presentation had no observable effect on copulation time (Figure 2). Overall copulation time (s) CI was 17–20 s; and average copulation time (s), CI and the total number of females sampled per cross-combination were MM-MF 17 (15–19, 24), MM-UF 19 (15–23, 24), UM-MF 18 (13–23, 26), UM-UF_CAM 21 (16–26, 26), and UM-UF_CC 19 (15–22, 32). The profile analysis revealed that the test within-subject effects is most compatible with no important effect of the order of female presentation to the male on the copulation time within the same cross-combination (F_8,38_ =2.075, *p* = 0.063). The test of between-subject effects or differences in the means between cross-combinations groups showed that there was not a significant difference in copulation time between the cross-combinations groups (across all three females) (F_1,19_ = 0.139, *p* = 0.966). The sphericity assumption, an assumption of equal variances in the differences between all possible pairs of within-subject conditions, was not violated (Mauchly’s Test of Sphericity X^2^ = 0.117, *p* = 0.943).

Insemination success was high across all treatments. Of the 132 females that copulated (four were not used for the analysis because they escaped or died before dissection), 91% were successfully inseminated. Almost 70% of the inseminated females had three spermathecae filled, and none of them had one (Table 1). Of the 80 females with three spermathecae filled, 34 of them (43%) had a low concentration of sperm in one of their lateral ones. The sperm was stored in the medial and lateral spermathecae in all females with two of them filled. Cross-combinations had no significant effect on the proportions of inseminated or non-inseminated females (*p* = 0.150), nor was a predictor of the number of filled spermathecae in inseminated females (F_4,108.5_ = 0.97, *p* = 0.429, R^2^ = 0.17). Of 11 females that did not copulate, five were presented first or second in sequence to a male that was able to copulate and inseminate with the next female.

#### 3.2.2. Mate Choice

To investigate if the presence or absence of fluorescent powders in females affects male sexual mate choice, a total of 30 unmarked virgin males (wing size 2.07 ± 0.06 mm) and 115 virgin females (wing size 2.71 ± 0.09 mm) were used. No significant variation in the female mean wing size between marked and unmarked females was detected by the ANOVA (F_1,113_ = 0.21, *p* = 0.885).

The presence or absence of powder in females had no observable effect on male mate choice. Proportions of inseminated or not inseminated females within each replicate and among both types of fluorescent powders (DG1 and DG2) were not significantly different (*p* = 0.388 and *p* = 0.094, respectively). No significant variation was found either in the proportions of inseminated or not inseminated females in marked or unmarked females (*p* = 0.162), and the proportions of inseminated marked or unmarked females with one, two, or three filled spermathecae (*p* = 0.128). More than 50% of the inseminated females had three spermathecae filled, and in all cases, the medial spermatheca was the first to be filled (Table 2).

### 3.3. Transference of Fluorescent Powders between Individuals during Copulation and Same-Sex Interactions

#### 3.3.1. Transference of Fluorescent Powders between Individuals during Copulation

For the mating process experiment, proportions of copulated females with transferred powder were not significantly different between combinations 1.MM-MF (88%) and 2.MM-UF (100%) (*p* = 0.234, N = 24). The least-squares regression (R^2^ = 0.59) to predict the female scores obtained due to copulation interaction revealed that only cross-combination (F _1,43_ = 12.22, *p* = 0.001) and female size (F _1,43_ = 4.82, *p* = 0.034) were significant predictors of the least squares mean (LSM) female powder score (Combination 1.MM-MF LSM = 1.99, SE = 0.27; combination 2.MM-UF LSM = 3.05 SE = 0.27). The relationship between the wing size and score was negative (t Ratio = −2.20), suggesting that larger females presented lower powder scores.

The transference of powder linked to copulation activity was concentrated in the areas of the genitalia, legs, and the tip of the wings and proboscis. Both groups of females, those used in the copulation time experiment and mate choice experiment, showed powder marking mostly in their genitalia, legs (score of two), and wings (score three). The rest of the score combinations included females with powder also in the proboscis and thorax. Almost all the males used in the mate choice experiment showed the powder in their genitalia, legs, and the tip of the wings and proboscis (Table 3).

#### 3.3.2. Transference of Fluorescent Powders between Same-Sex Individuals

**Figure 3:**
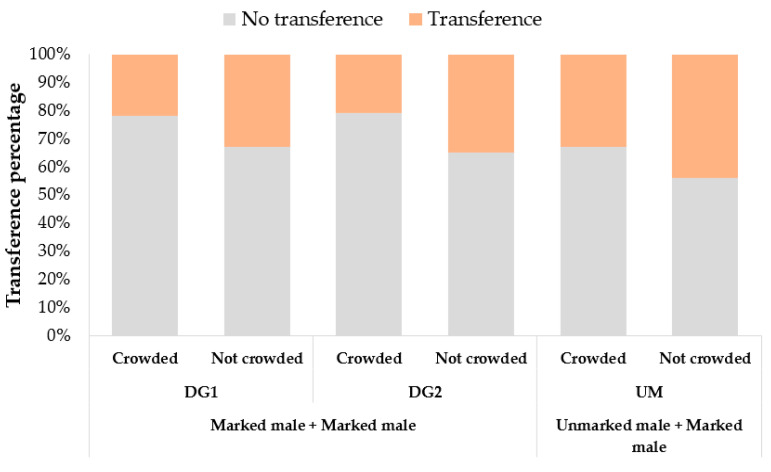
Transference of fluorescent powder (percentage) among *Ae. aegypti* males under crowded (N: 150 males) or not crowded (N: 30 males) conditions and different combinations (marked male + marked male, unmarked male + marked male). DG1 are marked males that received powder from a DG2 marked male, DG2 are marked males that received powder from a DG1 marked male, and UM are unmarked males that received powder from a DG1 marked male.

The transference of fluorescent powders between same-sex individuals happened in a similar way among both density conditions and combinations, but different amongst sexes. In the case of females, no evidence of transference of powder was observed, regardless of the combination or the density condition. In the case of males, transference was observed, but the proportions of males with transferred powder were not significantly different among density conditions C: 25%—NC: 28% (*p* = 0.49) or among combinations MM-MM/MM-UM (DG1 males that received powder from a DG2 marked male: 24%, DG2 males that received powder from a DG1 marked male: 23%, and, UM unmarked males that received powder from a DG1 marked male: 35%, *p* > 0.05) (Figure 3). Results of the interactions between unmarked female/male to marked female/male were not analyzed since no real evidence of the interaction can be registered. Proportions among replicates within each group were not significantly different (*p* = 0.521). An average of 74% of the males with transferred powder, exhibited the powder in legs and genitalia (DG1:74%, DG2:76%, UM:71%), while the rest of them in the legs, genitalia, and wings.

## 4. Discussion

Different descriptive aspects about the use of fluorescent powders in the multiple marking of mosquitoes are presented. This knowledge can help in decision making and defining their functionality when used as tools in different experimental settings. The limitations of using florescent powders can vary, depending on the detection system being used. For example, when human sight is the method for discriminating different colored powders, it is important to evaluate practical aspects of the experiment before committing to detailed protocols. Differences between experimental findings using powders can be linked to the use of different types of powders, powder concentration, method of application, and marked adult grooming behavior. Additionally, there are consistent reports of distinguishable combinations of orange with yellow, green, or blue and indistinguishable combinations of yellow–green [7,8]. The human capacity of distinguishing between colors is linked to the cone photoreceptors response to electromagnetic radiation with wavelengths between 400 to 700 nm (visible spectrum), and a trichromatic color vision enhanced by photoreceptors with wavelengths of maximal sensitivity in the yellow–green (around 560 nm), green (around 530 nm), and blue (around 430 nm) regions of the spectrum [39]. It is possible that when combining yellow and green powders together, since the light spectrum is continuous, this combination stimulates a group of photoreceptors that will respond to produce a sensation of seeing a color between green–yellow, and limit our capacity to perceive them as separate colors. This practical aspect will vary among types of powders because each of them will have their unique wavelengths absorption and reflection, so the exploration of other color mixtures is desirable. The use of violet fluorescent powder is not commonly reported, and despite the fact that in our case it was possible to recognize clusters of this powder in the mosquito body, it was also complex to differentiate it from mosquito white scales when illuminated with UV light, and when using two or more powders at the same time. Due to these difficulties, combinations using violet are not recommended if using a mosquito with white patterns.

In contrast to observations made in *Drosophila* adults, we found that the order of application had an effect in determining which powder was most easily detected, but not defining whether the two-powder combination was distinguishable from either powder alone [7]. It is possible that when applying the first powder, it covers much of the available body area and leaves less space for adherence of the second powder, and therefore the latter is less detectable. Previous experiments using the same powders, in a single application, have shown good mosquito body coverage and adherence that permitted their detectability until the final experimental day of observation in trials lasting 30 days [5]. This outcome is possibly aided by the interaction between the setose mosquito surface and cuticle electrostatic charge [40] with the polyamide/polyester copolymer, in which the pigments are mixed [3]. This aspect, coupled with the fact that more than two powders can be differentiated under the stereoscope, can be used as a tool for discriminating which was the first powder applied in a multiple marking sequence; but protocols for applying them at the adequate concentration and at different times must be optimized. Possible changes in the detectability of the powders applied in sequence (second to fifth) over time (more than 96 h), were not evaluated in our design and is a limitation of the research. Future studies that allow delineating the real temporal applicability of multiple marking are desirable. For practical purposes, research groups involved in multiple marking studies may be interested in developing a non-destructive detection system, able to give more consistency to the data collected by multiple groups, and for surpassing human eye limitations or individuals’ perception of color. This device could ideally be able to detect the emitted signal after the delivering UV light that excites the fluorescent powders present in the marked mosquitoes, and perceive different signals by using cameras, coupled magnifier lenses, filters or monochromators [41], and automated imaging software.

Regarding the impact of fluorescent powders in *Ae. aegypti* mating, a high percentage of copulation events, no difference among combinations in the coupling time, copulation time, insemination success nor mate choice, suggest low effects of these powders on the mentioned traits. Lack of studies evaluating this mating interaction in marked *Ae. aegypti* mosquitoes prevent us from establishing comparisons under similar conditions. Records of previous studies that have also used fluorescent powders, in both controls and treatments, for exploring *Ae. aegypti* copulation traits have demonstrated successful copulation and insemination in the control marked individuals [9,10].

Occurrence of copulation events scattered throughout couples of different ages and after successive copula, in both marked and unmarked individuals, is in accordance with a life-time *Ae. aegypti* natural mating state, ready to copulate when the proper stimulus is supplied [16]. Although not directly measured in our study design, the lack of impact of the powders in the ability of couples to find each other and be able to copulate, suggest that body parts related to the emission and reception of critical mating mediators (visual, acoustic, or chemical cues [11,12,16,17]) were not impeded in performing their task.

While comparisons of coupling time with other studies can be misleading due to differences in the methodology, the time registered in this experiment was similar to a previous report using *Ae. aegypti* unmarked males. In the mentioned study, the total time for single males to attempt or to successfully copulate ranged between 50 to 150 min, when using a single control marked male with five virgin female mosquitoes in successive copulation [10]. The same comparable results were also observed in relation to the copulation time, in which the range and mean obtained in this experiment, for all the cross-combinations, were similar to the ones reported in multiple previous experiments using unmarked *Ae. aegypti* males (range from 4 to 59 s, mean of 16 [16], 13.2 [20] and 13.7 s [21]). Our results also agree with male mating history not affecting mating duration [42] in a second or third sequential female mate.

A high percentage of insemination success in all marked or unmarked copulated females, as when analyzed in first, second, or third in the sequence, is consistent with reports of insemination rates in laboratory conditions of 74–100% using *Ae. aegypti* mosquitoes of different ages [11,34,43] and *Ae. aegypti* male capacity of inseminating 4–6 females consecutively [20,23,24]. Likewise, the high percentage of visualization of sperm in the three spermathecae in inseminated marked or unmarked females, agrees with other reports that allowed unmarked *Ae. aegypti* to copulate freely [20,34], but differs with others that report that usually one of the three spermathecae remains empty [16,44]. All experiments agreed that usually, the medial spermatheca is the first in being filled [16,20,34,44]. Discrepancies in results may be related to different methodologies, mainly at the moment of manipulating the spermathecae and observing the sperm using an optical device. Some experiments crushed the spermathecae to assure the presence of the sperm and recognized that it was difficult to decide whether sperm were originally present in that reservoir or whether they came from the other two crushed spermathecae [16]. In other experiments, the use of stereo microscopes, rather than compound microscopes, limits the observer capacity of recording low amounts of sperm in one of the lateral spermathecae. In our experience, the immediate dissection of recently anesthetized females and the observation of the spermathecae using 10× and 40× magnification in a compound microscope, allowed the visualization of circular cell movement of live spermatozoa [22] in low concentrations, and sometimes in the spermathecae that looked darker and less opaque that the evident filled ones. This last observation is important, since differences in spermathecae color, or its transparency, have been used as a practical approach for categorizing filled from not filled spermathecae under low magnifications. Although this observation is frequently coincidental, sometimes the spermatheca that look more translucent than the filled ones, also have a little amount of sperm.

The lack of male preference to a marked or unmarked female in the mate choice experiment, as well as no difference in the percentages of inseminated females in either group, suggests that the presence of fluorescent powders was not a barrier for mating decisions and ability to copulate and inseminate females. Future experiments can explore the effect of marked individuals in unmarked *Ae. aegypti* electroretinographs, since as it is already reported, females show a maxima spectral sensitivity in the ultraviolet and green wavelength ranges [45].

As part of the limitations of the study regarding the impact of fluorescent powders in *Ae. aegypti* mating, it is important to specify that the natural mating behavior of *Ae. aegypti* is rarely simulated in the laboratory and therefore, the conclusions from these experiments are condition dependent, i.e., apply only to circumstances similar to these laboratory protocols. Additionally, the female reproductive success after mating was not determined in marked females or unmarked females that copulated with a marked male.

In relation to the powder transference, we found evidence for asymmetric transference of fluorescent powders between same-sex interactions among mosquito sexes. The observation of transference solely between males, together with the lack of density effects, suggests that the mechanism is linked to male behavior. Observations of virgin males allowed us to record male–male interactions similar to male–female copulation contacts (Video S1). This behavior matched with the presence of powder in males in the same body regions as females after copulation. Similar observations of this behavior have been reported when large numbers of males are isolated from females, in which the frequency of males seizing same-sex conspecifics increase with the total number, and from old males to younger ones [16]. In our experiment, the number of males with transferred powder was not related to the density conditions used in our design, and although males were of similar age, the use of exactly the same age or exclusively virgin males were not controlled. It is possible that the percentage of transference may change modifying the mentioned variables, and when using this marking technique in field settings, where females will be available, and mating occurs mainly in flight swarming around the host [13]. These findings may be also useful in gauging mating rates in nature, between mosquitoes released as part of control programs evaluating the efficacy of mating success among released *Wolbachia*-infected *Ae. aegypti* [30] and RIDL (release of insect carrying a dominant lethal gene) *Ae. aegypti* with wild mosquitoes [31].

Powder transference in copulation was observed in the majority of females exposed to marked males. The presence of the powder predominantly in the legs and genitalia, and then in wings, is related to the *Ae. aegypti* mating behavior, that involves the male legs seizing specific parts of the female appendages during the coupling, and then the clasping of the tip of the female abdomen with his terminalia to insert his aedeagus into the genital chamber during the copulation [16]. Not all copulations occur in the first attempt, and sometimes when males and females are interacting, they need to reorient themselves or be more flexible, as is the case when the male initiates mating from the dorsal side of the female [43]. These latter contacts before the copulation may be related to the presence of the powder in the wings. The presence of the powder in the tip of the proboscis on almost all of the males suggest that they also interact physically with the female using this part of the head, possibly in the “venter-to-venter” position, that is how usual copulation occurs. Despite the fact that this interaction was recorded frequently, it is not essential for copulation, since males without a proboscis are able to locate females and copulate [16]. A higher powder score in unmarked females after exposition to a marked male, in comparison to already marked females, can be linked to the aforementioned observation of higher adherence and detection of the first powder that the mosquito comes in contact with. It is possible that females already marked, adhere less powder from males, than females with no previous powder in their cuticle. Additionally, fewer scores in large females may be related to previous reports suggesting that female body size is a major physical factor affecting female mating success [11], and in which larger virgin females are more likely to harmonically converge during a mating attempt than smaller females. This convergence is linked to fewer rejection behaviors towards the male and rather a single successful copula attempt [17]. It is possible that efficient copula leads to less physical interaction, and therefore the absence of powder in other female body regions not directly associated with copula, such as the wings.

Our findings are consistent with those found using *Ae. aegypti* [10], *An. arabiensis* [28], and *Psorophora* mosquitoes [26], where transference occurred from marked males to unmarked females, from marked males to both males and females, and from marked to unmarked individuals in a confined space, respectively. Differences in study findings, like a higher amount of powder transfer in *Ae. aegypti* [10], a lower percentage of transference in *Psorophora* mosquitoes [26], or the absence of transference in *Culicoides* [27] and in *Drosophila* [6] during mating, may be related to differences in the methodology, type and amount of powder, and species biology. From a practical perspective, it is important to specify that the transferred powder, between males or during copulation, was visible under high magnification and was restricted to the mentioned body areas, so although transference is possible, the amount and distribution of powder seen in a marked individual would potentially allow the differentiation of mosquitoes that were originally marked, from those who acquired it through transference. It has been proposed that a potential solution can include allowing the marked individuals to groom themselves before releasing them, placing them in a container that will also hold unmarked individuals, or allowing them to mix with the unmarked ones [6]. Although this action may reduce the amount of transferred powder, it is shown here that even after 24 h of allowing them to groom themselves before the interactions are permitted, transference still occurred.

## 5. Conclusions

The lack of evidence that the tested fluorescent powders affect *Ae. aegypti* coupling time, copulation time, insemination success, and mate choice, as well as the possibility of using them in studies that include multi-marking, make these powders a useful tool available for studies where those considerations are important. Recognition and attention of possible powder transference are necessary to avoid unnecessary bias. Future practical applications of these powders can be related to a trap development, in which the powder is administered automatically, and a sensor is able to record each marked individual that enters and exits the trap, eliminating all human handling. Additionally, due to the lack of effect of these powders on the several mosquito traits analyzed here, as well in past studies [4,5], other studies can explore the use of a similar resin in which these powders are mixed in for carrying slow-acting insecticides, entomopathogens, pheromones, among others, and potentially allowing for the secondary transfer of active ingredients to conspecifics during interactions or its delivery to oviposition sites [46].

## Figures and Tables

**Table 1:** Mean wing size (mm), insemination success and spermathecae filled for different cross-combinations of marked and unmarked *Aedes aegypti* males and females. LSM: least squares mean, SE: standard error.

**Cross-Combination**	**Mean Wing Size (mm)**		**Successful Insemination (%)**	**Number of Spermathecae Filled in Inseminated Females**			**Total Females**
	**Male**	**Female**		**2 (%)**	**3 (%)**	**LSM (SE)**	
MM-MF	2.07	2.75	23 (96)	6 (26)	17 (74)	2.72 (0.10)	24
MM-UF	2.07	2.75	20 (83)	10 (50)	10 (50)	2.52 (0.11)	24
UM-MF	2.08	2.68	21 (84)	7 (33)	14 (67)	2.67 (0.11)	25
UM-UF_CAM	2.06	2.75	23 (100)	4 (17)	19 (83)	2.80 (0.10)	23
UM-UF_CC	2.04	2.78	30 (94)	10 (33)	20 (67)	2.65 (0.09)	32
Total	2.06	2.74	117 (91)	37 (32)	80 (68)		128

**Table 2:** Inseminated females and spermathecae filled in marked and unmarked inseminated females after being exposed to males able to choose their mating partner.

**Female Condition**	**Inseminated Females (%)**	**Number of Spermathecae Filled in Inseminated Females (%)**			**Total Females**
		**1**	**2**	**3**	
Marked female	55 (96)	0	16 (29)	39 (71)	57
Unmarked female	51 (88)	1 (2)	22 (43)	28 (55)	58

**Table 3:** Powder score in females.

Score	Mating Process Experiment		Mate Choice Experiment	
	Copulated MF (%) Comb.1 MM-MF	Copulated UF (%) Comb.2 MM-UF	Inseminated UF (%)	Males (%)
0	3 (13)	0	10 (20)	0
1	4 (17)	2 (8)	3 (6)	0
2	6 (25)	5 (21)	14 (27)	0
3	10 (42)	8 (33)	15 (29)	0
4	1 (4)	8 (33)	8 (16)	29 (97)
5	0	1 (4)	1 (2)	1 (3)
Total (n)	24	24	51	30

## Supplementary Materials

The following are available online at https://www.mdpi.com/2075-4450/11/11/727/s1, Video S1: Males interaction.
